# Early apixaban administration considering the size of infarction and functional outcome in acute ischemic stroke

**DOI:** 10.3389/fneur.2024.1302738

**Published:** 2024-01-26

**Authors:** Min Hwan Lee, Jaseong Koo, Hanim Kwon, Jun Young Chang, Dong-Wha Kang, Sun U. Kwon, Jong S. Kim, Bum Joon Kim

**Affiliations:** ^1^Department of Neurology, Seoul St. Mary’s Hospital, University of Catholic, Seoul, Republic of Korea; ^2^Department of Neurology, Korea University Ansan Hospital, Korea University College of Medicine, Ansan, Republic of Korea; ^3^Department of Neurology, Asan Medical Center, University of Ulsan College of Medicine, Seoul, Republic of Korea; ^4^Department of Neurology, Gangneung Asan Hospital, University of Ulsan College of Medicine, Gangneung, Republic of Korea

**Keywords:** NOAC, apixaban, acute stroke treatment, atrial fibrillation, prognosis

## Abstract

**Background and purpose:**

Atrial fibrillation-related stroke (AF-stroke) is associated with an adverse prognosis, characterized by a high incidence of progression, recurrence, and hemorrhagic transformation. Our study aims to investigate the potential benefits of stratified early administration of apixaban, taking into account infarct size during the acute phase, in order to enhance functional outcomes.

**Methods:**

We conducted this study at a tertiary referral stroke center, enrolling acute AF-stroke patients who received apixaban during the acute phase. Infarct size was categorized as small, medium, or large based on diffusion-weighted imaging. Patients were divided into two groups: standard initiation (apixaban initiation based on guidelines, i.e., small: 4 days, medium: 7 days, large: 14 days after stroke) and early initiation (initiation before guideline recommendations) groups. We compared favorable outcomes (modified Rankin scale score ≤ 2) at 3 months post-stroke, stroke progression, early recurrence, and symptomatic hemorrhagic transformation (sHT) between the groups.

**Results:**

Out of 299 AF-stroke patients, 170 (56.9%) were in the early initiation group. A favorable outcome was observed in 105 (61.8%) patients in the early initiation group and 62 (48.1%) patients in the standard initiation group (*p* = 0.019). Stroke progression or early recurrence occurred less frequently in the early initiation group (4.7% versus 13.2%, *p* = 0.007). Nevertheless, no difference in sHT was noted between the groups. Early initiation of apixaban was independently associated with favorable outcomes (odds ratio: 2.75, 95% confidence interval: 1.44–5.28, *p* = 0.002).

**Conclusion:**

Our findings suggest that early initiation of apixaban, tailored to infarct size, could serve as a viable strategy to enhance functional outcomes. This approach may potentially decrease stroke progression and early recurrence without elevating the risk of sHT.

## Introduction

Atrial fibrillation-related ischemic stroke (AF-stroke) is associated with an unfavorable prognosis compared to other stroke causes (1, 2), characterized by a heightened risk of progression, hemorrhagic transformation (HT), and early recurrent ischemic stroke (ERIS) (3–8). Nevertheless, optimizing antithrombotic treatment for enhanced AF-stroke prognosis remains a challenge. Early conventional anticoagulation initiated within 14 days of onset has demonstrated efficacy in reducing thrombotic events, yet the balance tilts due to an elevated bleeding risk, resulting in limited net clinical benefit (9).

In the current era of Non-Vitamin K Oral Anticoagulants (NOACs), guidelines advocate for a tailored approach to NOAC administration, taking into account specific stroke characteristics such as the severity of neurological deficit or infarction size (10, 11). Recent large-scale Randomized controlled trials like ELAN (Early versus Later Anticoagulation for Stroke with Atrial Fibrillation) and TIMING (Timing of Oral Anticoagulant Therapy in Acute Ischemic Stroke With Atrial Fibrillation) have reaffirmed the safety of early NOAC utilization and indicated a potential reduction in the recurrence of ischemic stroke (12, 13). Nonetheless, the effects of initiating NOACs early after acute ischemic stroke on functional outcomes still lack clarity.

Apixaban, validated for its efficacy in mitigating both ischemic and hemorrhagic events (14, 15), presents a promising avenue for potential advantages when administered early post-stroke. Consequently, this study aims to investigate whether the early initiation of apixaban, in contrast to administration guided by infarct size and in accordance with established guidelines, could exert an impact on functional outcomes, ERIS, and the progression of stroke in individuals suffering from AF-stroke.

## Methods

### Subjects

Patients experiencing acute ischemic stroke and admitted to the stroke center at Asan Medical Center from January 2017 to July 2020 were subjected to initial screening. Sequentially, individuals meeting the criteria for acute ischemic stroke were included in the study if they met the following conditions: (1) exhibited non-valvular atrial fibrillation warranting long-term anticoagulation and (2) initiated treatment with apixaban during their hospitalization. Exclusion criteria encompassed patients commencing anticoagulation with other agents, transitioning to apixaban, discontinuing anticoagulation, or lacking 3-month functional outcome data.

Routine management adhered to local guidelines, and data were collected through a prospective stroke registry. Cardiac assessment involving a 12-lead electrocardiogram, telemonitoring in the stroke unit, Holter monitoring, and transthoracic echocardiography were gleaned from medical records. Functional outcomes were evaluated at the 3-month mark. The study received ethical approval from the Asan Medical Center institutional review board (S2021-0483-0001), with the requirement for written informed consent waived due to the retrospective nature of the study.

### Neuroimaging and size of infarction

Neuroimaging was conducted at the emergency medical center within an hour of the patient’s arrival. The dimensions of the primary infarcts were ascertained through analysis of diffusion-weighted images (DWIs). Using the embedded software of our picture archiving and communication system, the maximum length of the infarct was measured employing a digital caliper.

The infarct size was stratified into the following categories: (1) small (maximum diameter ≤ 1.5 cm within the anterior or posterior circulation), (2) medium (involving infarction within the superficial cortical branch of the middle cerebral artery [MCA], the MCA deep branch, the internal border zone territories, or a superficial cortical branch of the posterior or anterior cerebral artery [PCA or ACA]), and (3) large infarcts (encompassing infarctions spanning the entirety of the MCA, PCA, or ACA territories, or occurring within two superficial cortical branches of the MCA, a superficial cortical branch of MCA concomitant with the MCA deep branch, or within less than one artery territory). Brainstem or cerebellum lesions exceeding 1.5 cm were classified as large infarcts (4).

The evaluation and measurement of brain imaging were executed by a proficient radiologist and a vascular neurologist, both of whom were blinded to all clinical data.

### Initiation of anticoagulation

The timing of anticoagulant initiation was at the discretion of the attending physician, who made decisions based on the patient’s specific medical information. In the absence of robust evidence in the literature, physicians may vary regarding the criteria and opinions on the optimal timing for initiating anticoagulation therapy.

The conventional initiation of apixaban adhered to the parameters outlined in the European Stroke Organization and Karolinska Stroke Update 2016 guidelines, stipulating commencement at 4, 7, and 14 days following the onset of stroke for instances of small, medium, and large infarcts, respectively (11). Early initiation, on the other hand, referred to the commencement of anticoagulation prior to the aforementioned guideline-recommended timeframes. This encompassed initiation within 3, 6, and 13 days after the onset of stroke for cases of small, medium, and large infarcts, respectively.

### Outcomes

The primary endpoint of this study focused on determining the proportion of patients displaying favorable functional outcomes 3 months subsequent to their ischemic stroke. The attainment of a favorable outcome was defined as a modified Rankin scale (mRS) score of 2 or lower. The assessment of the mRS score was conducted either during a routine outpatient visit or via telephone, employing a structured interview facilitated by a trained nurse.

Secondary outcomes included the evaluation of stroke progression, ERIS, symptomatic HT (sHT), and mortality. Stroke progression was characterized by deterioration in the neurological deficit, indicated by a National Institute of Health Stroke Scale (NIHSS) score of 2 or higher during hospitalization, with confirmation of infarct growth through follow-up DWI. ERIS was defined as the emergence of a new neurological deficit or an NIHSS score of 2 or greater, accompanied by a distinct DWI lesion separated from the initial DWI lesion, and confirmed within 3 months from the onset of the index stroke. sHT referred to clinical deterioration defined by an increase of more than 4 points of NIHSS score. Instances of hemorrhage preceding apixaban initiation were excluded from consideration. Additionally, mortality events within 1 year were documented.

### Statistical analysis

Patients with early initiation of anticoagulation (early apixaban group) and standard initiation (standard apixaban group) were compared. Statistical comparisons were carried out between these groups. Discrete variables were subjected to Pearson’s chi-squared test or Fisher’s exact test, while continuous variables were analyzed using either the Student’s *t*-test or analysis of variance (ANOVA), as deemed appropriate.

For examining factors associated with the favorable outcome at the 3-month mark, both univariable and multivariable analyses were conducted. Initially, univariable analysis was employed, and factors displaying clinical relevance or demonstrating potential associations (with a significance level of *p* < 0.10) were incorporated into the multivariable analysis model for favorable outcomes. All statistical tests were two-tailed, and the threshold for statistical significance was defined as *p* < 0.05. The statistical analyses were executed utilizing R software (version 4.0.4; R Foundation, Vienna, Austria).

## Results

**Figure 1:**
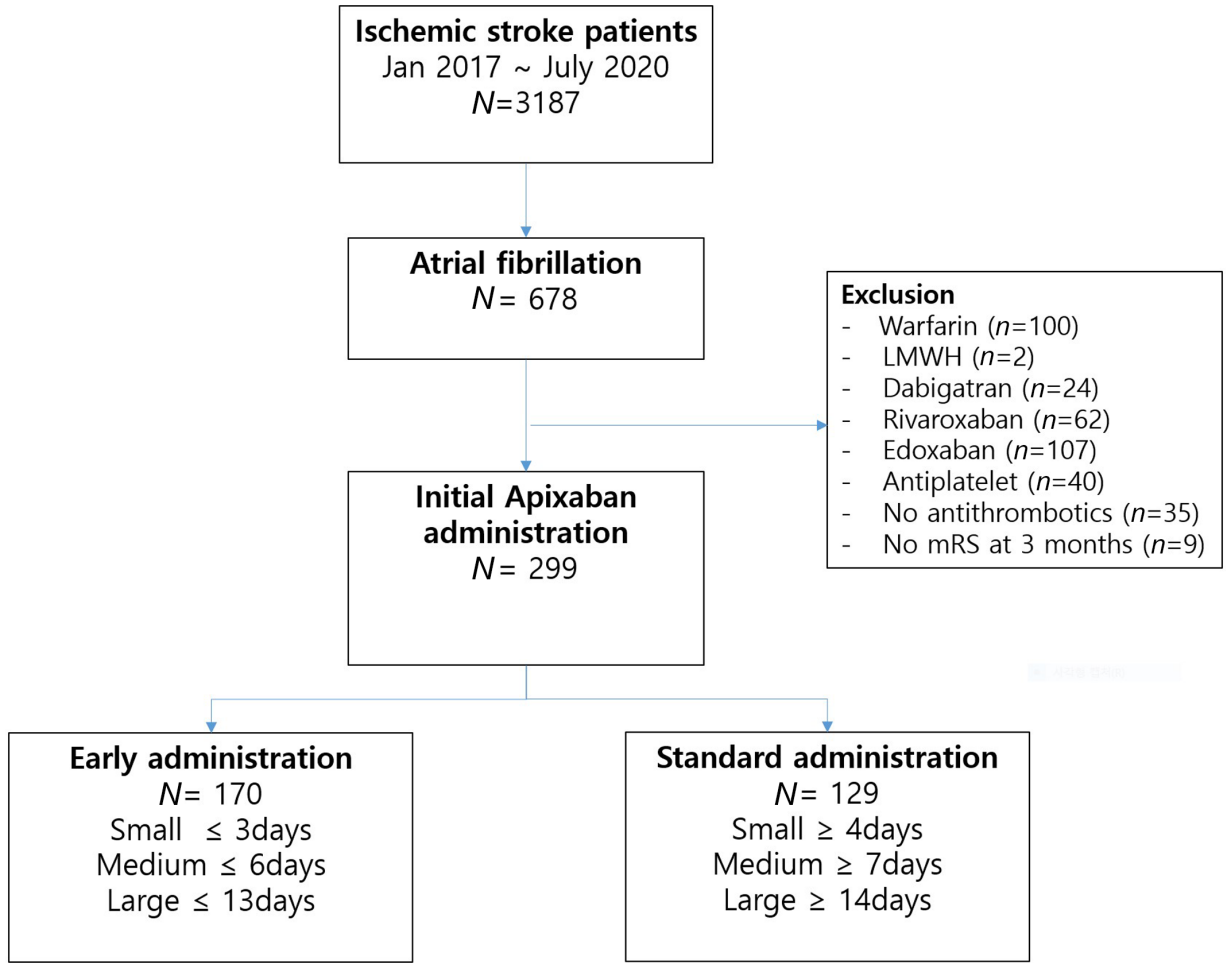
Flow chart of the study patients. N, number; LMWH, low-molecular-weight heparin; mRS, modified Rankin scale score.

**Figure 2:**
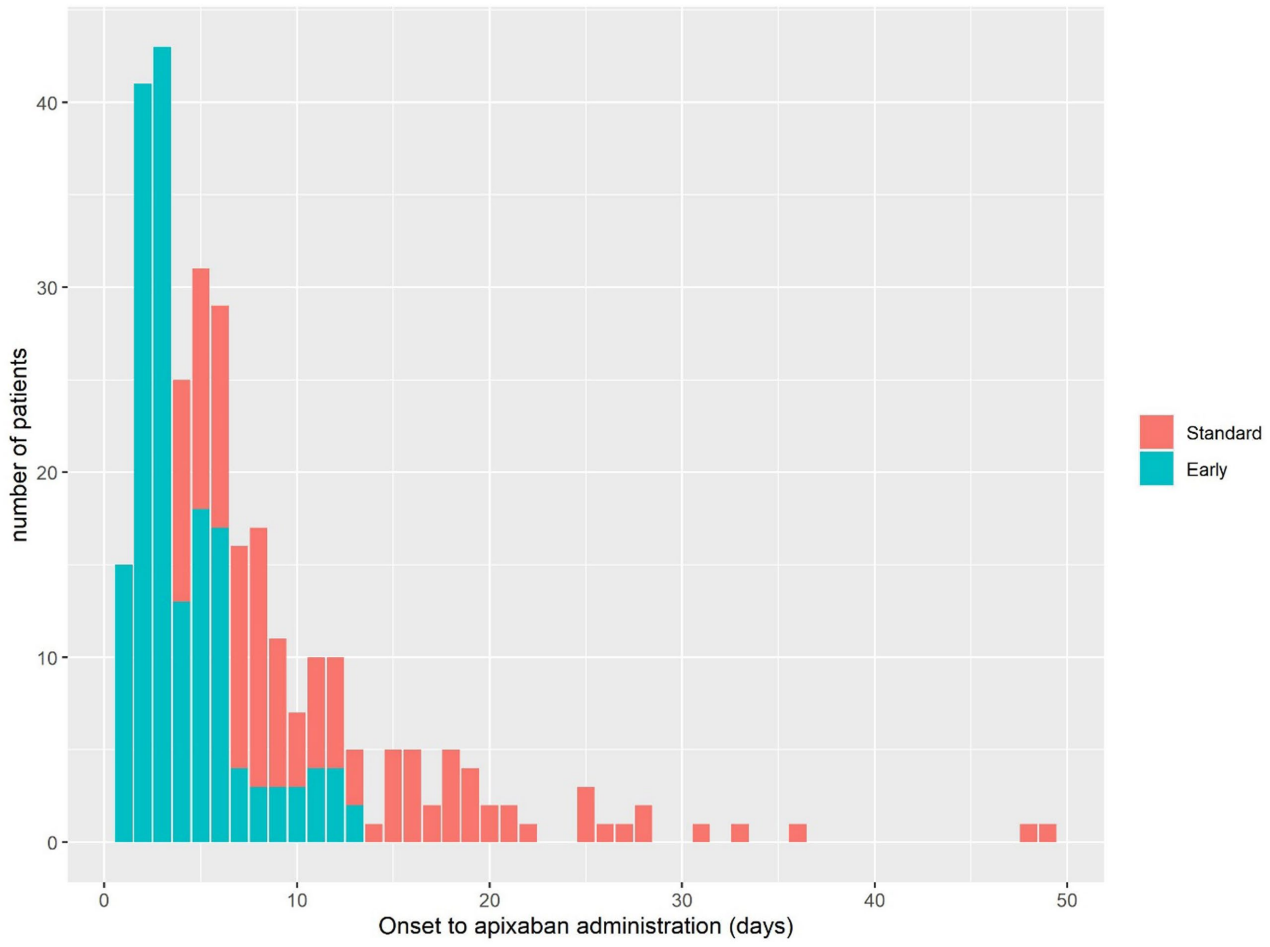
Timing of apixaban administration after AF-stroke.

Throughout the study duration, a total of 3,187 patients were admitted due to ischemic stroke. Among these cases, 678 patients (21.3%) exhibited non-valvular AF. Among the AF patients, apixaban was administered to 308 individuals (45.4%); however, nine patients lacking 3-months mRS data were subsequently excluded. Ultimately, the final analysis comprised 299 patients (Figure 1). The initiation of apixaban occurred at a median interval of 5 days (with an interquartile range [IQR] of 3–9 days) following the onset of stroke, shown in Figure 2. Notably, a favorable outcome was observed in 167 patients (55.9%).

### Early vs. standard apixaban initiation

The baseline characteristics of the patient cohort are presented in Table 1. The average age of the patients was 73.4 ± 9.9 years, with 124 individuals (41.5%) being female. Among the participants, a total of 170 patients (56.9%) were classified within the early initiation group.

**Table 1:** Baseline characteristics in early and standard apixaban groups.

	Early apixaban	Standard apixaban	*p* value
	(*N* = 170)	(*N* = 129)	
Female	72 (42.4)	52 (40.3)	0.813
Age, years	73.9 ± 10.0	72.8 ± 9.9	0.371
Hypertension	117 (68.8)	89 (69.0)	1.000
Diabetes	50 (29.4)	36 (27.9)	0.876
Dyslipidemia	59 (34.7)	38 (29.5)	0.403
Smoking	48 (28.2)	44 (34.1)	0.335
Previous stroke/TIA	52 (30.6)	32 (24.8)	0.331
Previous antithrombotics	72 (42.4)	49 (38.0)	0.520
CHA2DS2-VAS_C_ score	5.0 (4.0–6.0)	5.0 (4.0–6.0)	0.663
HAS-BLED score	3.0 (2.0–4.0)	3.0 (2.0–4.0)	0.868
CrCl, ml/min	75.0 (58.0–87.0)	74.0 (57.0–87.0)	0.788
D-dimer, mcg/ml	0.8 (0.4–2.0)	0.9 (0.5–2.6)	0.377
Echocardiographic findings^†^			
Left atrium size, mm	45.0 (41.0–49.0)	45.0 (39.0–49.0)	0.410
LVEF, %	60 (56–64)	60 (54–64)	0.623
LV wall motion abnormality (%)	32 (18.9)	26 (20.5)	0.403
Other potential source of cardioembolism^‡^	7 (4.1)	7 (5.4)	0.799
Stroke lesion size			0.003
Small	53 (31.2)	65 (50.4)	
Medium	80 (47.1)	41 (31.8)	
Large	37 (21.8)	23 (17.8)	
HT upon admission	24 (14.1)	14 (10.9)	0.507
IVT	24 (14.1)	16 (12.4)	0.795
IAT	28 (16.5)	26 (20.2)	0.504
IVT and IAT	5 (2.9)	5 (3.9)	0.904
NIHSS score	5 (2.0–11.0)	6 (2.0–14.0)	0.377
Onset to first apixaban administration, days	3 (2–5)	9 (6–16)	<0.001
Apixaban dose			0.952
2.5 mg	105 (61.8)	81 (62.8)	
5.0 mg	65 (38.2)	48 (37.2)	
Labeling dose			0.163
Label standard	65 (38.2)	48 (37.2)	
Label low-dose	37 (21.8)	18 (14.0)	
Off-label low-dose	68 (40.0)	63 (48.8)	

Values are given as mean ± SD, no. (%), or median (interquartile range). TIA, transient ischemic attack; CrCl, creatine clearance; LV, left ventricle; LVEF, left ventricular ejection fraction; HT, hemorrhagic transformation; IVT, intravenous thrombolysis; IAT, intra-arterial thrombectomy; NIHSS, National Institute of Health Stroke Scale.

† 296 patients underwent transthoracic echocardiogram.

‡ Left atrial appendage (LAA) thrombus (*n* = 1), patent foramen ovale (PFO) (*n* = 4), left atrial spontaneous echo contrast (*n* = 3), rheumatic mitral stenosis (*n* = 4), moderate aortic stenosis (*n* = 1) and dilated cardiomyopathy (*n* = 1).

Patients in the early and standard initiation groups received apixaban at a median interval of 3 days (with an IQR of 2–5 days) and 9 days (with an IQR of 6–16 days) after the onset of the index stroke, respectively. Notably, no significant disparities were noted in demographic variables, encompassing prior stroke history, risk factors, laboratory findings, reperfusion treatments, and cardiac evaluations, between the two groups. However, the early initiation group exhibited a higher proportion of medium or large stroke sizes compared to the standard initiation group. Nevertheless, no distinctions were evident in the initial stroke severity between the two groups (Table 1).

**Figure 3:**
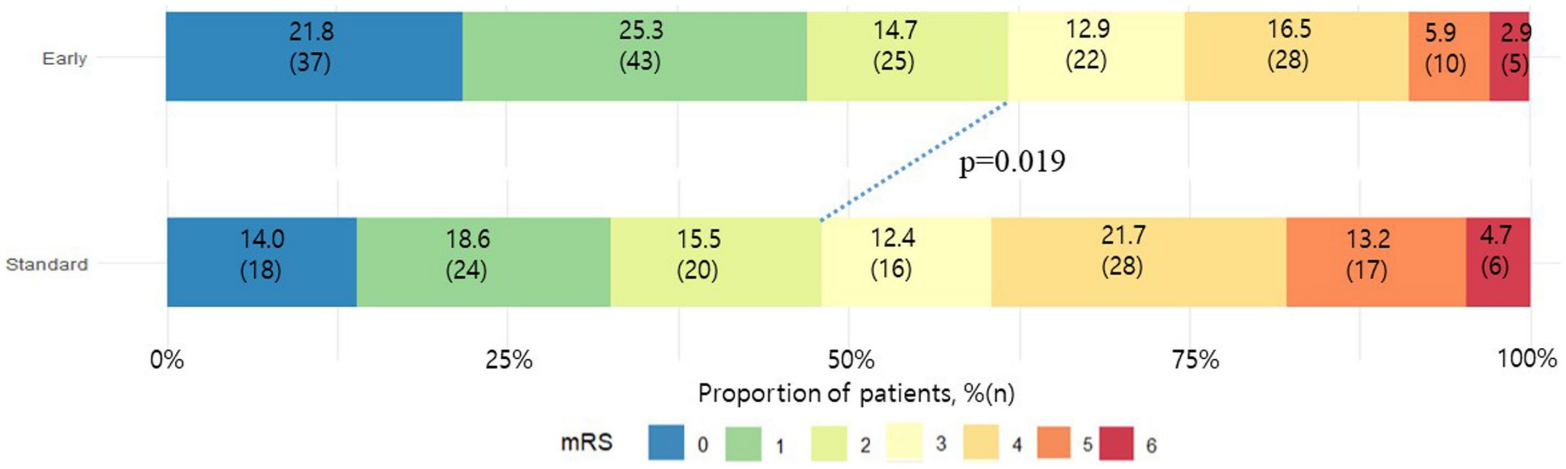
mRS scores at 3 months for the early and standard anticoagulation groups.

In terms of outcomes, a favorable functional outcome was witnessed in 105 patients (61.8%) within the early initiation group, while 62 patients (48.1%) within the standard initiation group achieved a favorable outcome (odds ratio [OR], 1.75 [95% confidence interval [CI], 1.10–2.78]; *p* = 0.019; adjusted OR, 2.75 [95% CI, 1.44–5.28]; *p* = 0.002), as shown in Figure 3. Additionally, within the early initiation group, the incidence of ERIS (2.4% versus 7.0%, *p* = 0.018), stroke progression (2.4% versus 6.2%, *p* = 0.015), and the combined occurrence of stroke progression and ERIS (4.7% versus 13.2%, *p* = 0.007) were significantly lower compared to the standard initiation group. However, no significant difference was observed in the proportion of patients experiencing sHT between the two groups (Table 2).

**Table 2:** Univariable and multivariable analyses: primary and secondary outcomes for apixaban initiation time.

Parameters	Apixaban start timing, *n* (%)		Unadjusted analysis		Adjusted analysis^†^	
	Early	Standard				
	(*N* = 170)	(*N* = 129)	OR (95% CI)	*p* value	OR (95% CI)	*p* value
Primary functional outcome at 3 months						
Favorable outcome	105 (61.8)	62 (48.1)	1.75 (1.10–2.78)	0.019	2.75 (1.44–5.28)	0.002
Secondary outcome						
ERIS in 3 months	4 (2.4)	9 (7.0)	0.32 (0.10–1.07)	0.064	0.15 (0.03–0.72)	0.018
Stroke progression	4 (2.4)	8 (6.2)	0.36 (0.11–1.24)	0.106	0.13 (0.02–0.67)	0.015
Stroke progression or ERIS	8 (4.7)	17 (13.2)	0.33 (0.14–0.78)	0.012	0.13 (0.04–0.42)	0.007
sICH in 3 months	2 (1.2)	1 (0.8)	1.52 (0.14–16.9)	0.732	1.95 (0.15–26.01)	0.613
Death in 1 year	9 (5.3)	8 (6.2)	0.85 (0.32–2.26)	0.737	0.80 (0.28–2.32)	0.680

ERIS, early recurrent ischemic stroke; sICH, symptomatic intracranial hemorrhage; OR, odds ratio and CI, confidence interval.

† Adjusted for age, gender, smoking, diabetes, previous stroke history, D-dimer, intra-arterial thrombectomy, hemorrhagic transformation at index image, stroke lesion size, apixaban dose, and NIHSS at admission.

### Factors associated with favorable functional outcome

Through univariable analysis, several variables demonstrated associations with functional outcomes. These variables encompassed age, diabetes, smoking status, previous history of stroke/TIA, CHA2DS2-VASC Score, D-dimer levels, size of the ischemic stroke lesion, undergoing intra-arterial thrombectomy (IAT), presence of HT upon admission, initial NIHSS score, and the early initiation of apixaban (Supplementary Table 1).

In the context of multivariable analysis, several factors exhibited independent associations with favorable outcomes. These factors included a previous history of stroke (OR, 0.47 [0.24–0.92]; *p* = 0.027), initial NIHSS score (OR, 0.81 [0.76–0.87]; *p* < 0.001), large lesion size (OR, 0.24 [0.09–0.65]; *p* = 0.005), and early initiation of apixaban (OR, 2.75 [1.44–5.28]; *p* = 0.002), as detailed in Tables 2, 3.

**Table 3:** Factors associated with favorable functional outcome.

	Unadjusted analysis	*p* value	Adjusted analysis^†^	*p* value
Parameters	OR (95% CI)		OR (95% CI)	
Male (Ref., Female)	1.59 (1.00–2.52)	0.052	1.19 (0.57–2.46)	0.642
Age, years	0.96 (0.94–0.98)	<0.001	0.99 (0.95–1.02)	0.406
Diabetes	0.52 (0.31–0.86)	0.01	0.54 (0.28–1.05)	0.069
Smoking	1.87 (1.12–3.13)	0.016	1.36 (0.61–3.02)	0.457
Previous stroke/TIA history	0.48 (0.29–0.80)	0.005	0.47 (0.24–0.92)	0.027
Intra-arterial thrombectomy	0.39 (0.21–0.72)	0.003	2.42 (0.91–6.46)	0.078
D-dimer	0.85 (0.76–0.94)	0.002	0.91 (0.81–1.02)	0.094
Stroke lesion size				
Small	1		1	
Medium	0.43 (0.25–0.74)	0.002	0.85 (0.41–1.76)	0.659
Large	0.14 (0.07–0.27)	<0.001	0.24 (0.09–0.65)	0.005
HT at admission	0.47 (0.23–0.94)	0.032	0.72 (0.28–1.88)	0.508
NIHSS at arrival	0.82 (0.78–0.86)	<0.001	0.81 (0.76–0.87)	<0.001
Apixaban dose, 5 mg (Ref., 2.5 mg)	4.13 (2.45–6.95)	<0.001	1.95 (0.97–3.91)	0.061

OR, odds ratio; CI, confidence interval; Ref., reference group; TIA, transient ischemic attack; HT, hemorrhagic transformation; NIHSS, National Institute of Health Stroke Scale.

† Adjusted for age, gender, smoking, diabetes, previous stroke history, D-dimer, intra-arterial thrombectomy, hemorrhagic transformation at index image, stroke lesion size, apixaban dose, and NIHSS at admission.

In the subgroup analysis stratified by the size of cerebral infarction, the early administration of apixaban was linked to a favorable outcome at the three-month mark when compared to standard initiation. This trend was notable among patients with medium or large lesions (medium lesion: 63% versus 37%, *p* = 0.012; large lesion: 38% versus 9%, *p* = 0.03). However, no statistically significant distinction in favorable outcomes was observed in patients with small lesions (77% versus 69%, *p* = 0.436).

### Subgroup according to lesion size

Patients with different lesion sizes (small, medium, and large) received apixaban at varying intervals after the index stroke: 4 days [with an IQR of 3–6 days], 5 days [with an IQR of 3–8 days], and 11 days [with an IQR of 7–18 days], respectively (*p* < 0.001). Interestingly, a higher incidence of lower and off-label apixaban doses was observed as the stroke lesion size increased (*p* < 0.001, Supplementary Table 2). The occurrence of stroke progression also demonstrated an upward trend with increasing lesion size (*p* < 0.001). In contrast, the rate of sHT following apixaban exhibited no significant escalation with an increase in lesion size (*p* = 0.847, Supplementary Table 2).

## Discussion

The data analysis indicates that the early initiation of apixaban, while taking into account the size of the infarction, is independently linked to a favorable outcome. The observed favorable functional outcome can potentially be attributed to the impact of early initiation, leading to a reduction in the occurrences of ERIS and stroke progression, all the while not exacerbating the incidence of sHT. Notably, the propensity of early initiation to diminish functional independence was particularly noteworthy in individuals with medium or large lesions.

In this study, a favorable prognosis at the 3-month mark was observed in 55.9% of cases. This falls within the range of 40–72% (16–18), as previously demonstrated in existing research, making our findings comparable. While most prior studies on early anticoagulation primarily assessed the risk of ERIS and sHT, our study evaluated the mRS score at the 3-month mark. Considering the potential impact of anticoagulant use on the severity of subsequent ischemic strokes and early neurological deterioration (19, 20), focusing solely on the rate of ERIS might lead to an underestimation of the effectiveness of early anticoagulation. Nonetheless, all of these factors are encompassed within the mRS score assessment.

A previous observational study has linked early anticoagulation to favorable outcomes (18). However, these studies often involved early treatment groups with lower stroke severity and mixed warfarin patient populations, leading to less definitive results. Notably, ELAN study (12), employing a similar anticoagulation approach, did not find a significant improvement in functional outcomes with early NOAC imitation. ELAN enrolled patients with low stroke severity, potentially excluding those with larger stroke cases with early progression. Furthermore, our study highlights anticoagulation initiation time differences, especially in larger strokes. We observed a delayed administration of later-initiated anticoagulants, more pronounced in larger strokes (Our study vs. ELAN, early group: 2, 4, 8 days vs. 2, 2, 6 or 7 days; standard group: 6, 10, 19 days vs. 3 or 4, 6 or 7, 12 or 13 or 14, respectively for small, medium, and large infarction). This is crucial, as larger infarctions carry heightened risks of ischemic events (16).

Stroke progression, along with ERIS, contributes significantly to early neurological deterioration and poor functional outcomes (3). Thrombus extension and distal re-embolism play a vital role, and early NOAC use might be effective in preventing these events. Large observational studies have identified early anticoagulation as a robust factor linked to decreased early neurological deterioration (20). Animal experiments have shown that apixaban effectively inhibits brain thrombin activity, reducing the final infarct size (21). In our study, the early NOAC group exhibited significantly lower rates not only of RIS but also of stroke progression.

Moreover, larger infarctions carry a heightened potential risk of sHT (4, 22). In a previous observational study, the early use of apixaban within 2 days in AF stroke with concurrent large vessel occlusion demonstrated comparable safety to later use (23). However, dosing considerations arise for early apixaban initiation in patients with medium to large lesions. The prevalence of low-dose apixaban usage was notably high among those with large infarctions, potentially due to the acute-stage sHT risk. A prior study indicated a trend of fewer major bleeding events with off-label low-dose NOACs (24). However, prolonged off-label low-dose apixaban use correlated with an elevated stroke recurrence risk (25). Therefore, adhering to labeled dosing becomes crucial once the sHT risk decreases in the subacute-to-chronic phase.

Interestingly, this study was unable to identify significant factors associated with the timing of apixaban administration. The groups initiating anticoagulation early and those following the standard initiation protocol showed similar baseline characteristics, including CHA2DS2-VASC and HAS-BLED scores. In a survey, only 36% of practitioners adhere to the ESC’s ‘1–3–6-12’ rule for anticoagulant administration timing, with even greater uncertainty in cases of moderate stroke (26). Our study also observed the highest proportion of early anticoagulation therapy initiation among patients with medium-sized stroke lesions in line with these findings, the variability of clinical practices in a real-world setting.

Our study has several limitations. This study is retrospective in nature, and while premorbid mRS was not collected, key factors such as previous stroke history, initial stroke severity were well balanced between the two groups. However, differences could still exist due to unmeasured variables like AF burden. The infarct size groups were categorized using a semi-quantitative methodology, and differences in infarct volume within each group were not evaluated. Furthermore, observational design study warrants careful interpretation. Second, early NOAC usage demonstrated relatively greater efficacy in cases of large infarctions. However, in this study, a group that could not receive inpatient NOAC initiation (e.g., large hemispheric infarction, early symptomatic HT, significant mass effect) was not analyzed. The TIMING study indicated that early NOAC administration could potentially be detrimental in severe ischemic stroke (NIHSS >15) (13). Third, the scope of this research was confined to the investigation of apixaban, which constrains the extrapolation of the results to other NOACs. Finally, interethnic differences could influence the acute ischemic stroke treatment effect of early NOAC usage. In the Practical “1–2-3-4-Day” Rule study, which considered NIHSS severity of strokes, early NOAC use significantly reduced ERIS in Asians, while the effect wasn’t significant in Western populations (27). Additionally, within the ELAN study, the effect of early NOAC use was even more pronounced in Japan (12). This study is also limited to the Korean population. The racial differences in the effectiveness of anticoagulants warrant further investigation.

In conclusion, this study showed that differentiated early apixaban administration timing, considering the size of infarction, was associated with better functional outcomes at 3 months compared to standard administration. Early apixaban treatment showed a reduction of ERIS and stroke progression and no increase in sHT.

## Data availability statement

The raw data supporting the conclusions of this article will be made available by the authors, without undue reservation.

## Ethics statement

The studies involving humans were approved by the Asan Medical Center Institutional Review Board (S2021-0483-0001). The studies were conducted in accordance with the local legislation and institutional requirements. The ethics committee/institutional review board waived the requirement of written informed consent for participation from the participants or the participants’ legal guardians/next of kin due to the retrospective nature of the study.

## Author contributions

ML: Conceptualization, Formal analysis, Methodology, Writing – original draft. JK: Supervision, Writing – review & editing. HK: Data curation, Writing – review & editing. JC: Data curation, Writing – review & editing. D-WK: Data curation, Writing – review & editing. SK: Data curation, Writing – review & editing. JK: Data curation, Writing – review & editing. BK: Conceptualization, Data curation, Funding acquisition, Methodology, Writing – review & editing.
