# Supplementary material for: Early apixaban administration considering the size of infarction and functional outcome in acute ischemic stroke
Source: Front Neurol. 2024 Jan 26;15:1302738. doi: 10.3389/fneur.2024.1302738 (PMC10853473; doi:10.3389/fneur.2024.1302738)
Supplement: Supplementary file 1 [file Table_1.DOCX]

**Supplementary1. Univariate analysis for favor functional outcome at 3 month**

|  | Poor functional outcome  (n=132) | Favor functional outcome  (N=167) | P |
| --- | --- | --- | --- |
| Female | 63 (47.7) | 61 (36.5) | 0.067 |
| Age ,years | 75.6 ± 10.5 | 71.6 ± 9.1 | 0.001 |
| Hypertension | 95 (72) | 111 (66.5) | 0.371 |
| Diabetes | 48 (36.4) | 38 (22.8) | 0.014 |
| Dyslipidemia | 45 (34.1) | 52 (31.1) | 0.676 |
| Smoking | 31 (23.5) | 61 (36.5) | 0.021 |
| Previous stroke/TIA history | 48 (36.4) | 36 (21.6) | 0.007 |
| Previous antithrombotic medication | 59 (44.7) | 62 (37.1) | 0.228 |
| CHA2DS2-VASc Score | 5.0 (4.0-6.0) | 5.0 (4.0-6.0) | <0.001 |
| Onset to arrival delay,<6hr | 54 (41.9) | 75 (46.9) | 0.463 |
| Lab findings |  |  |  |
| CrCl, ml/min | 74.0 (58.0-88.5) | 76.0 (58.0-86.0) | 0.636 |
| D-dimer, mcg/dl | 1.5 (0.6-3.3) | 0.6 (0.4-1.2) | <0.001 |
| Echocardiographic findings* |  |  |  |
| Left atrium size, mm | 45 (39-49) | 45 (40-50) | 0.269 |
| LVEF, % | 60 (56-64) | 60 (55-64) | 0.907 |
| LV Wall motion abnormality | 23 (17.8) | 35 (21) | 0.774 |
| Stroke lesion size |  |  | <0.001 |
| Small | 32 (24.2) | 86 (51.5) |  |
| Medium | 56 (42.4) | 65 (38.9) |  |
| Large | 44 (33.3) | 16 (9.6) |  |
| IV thrombolysis | 18 (13.6) | 22 (13.2) | 1.000 |
| IA thrombectomy | 34 (25.8) | 20 (12.0) | 0.003 |
| IV and IA thrombolysis | 8 ( 6.1) | 2 ( 1.2) | 0.046 |
| HT at admission | 23 (17.4) | 15 (9.0) | 0.045 |
| NIHSS severity |  |  | <0.001 |
| Mild† | 36 (27.3) | 137 (82.0) |  |
| Moderate‡ | 57 (43.2) | 20 (12.0) |  |
| Severe§ | 39 (29.5) | 10 ( 6.0) |  |
| Onset to apixaban delay, days | 8.0 (4.0-15.0) | 4.0 (3.0- 6.5) | <0.001 |
| Early apixaban administration | 65 (49.2) | 105 (62.9) | 0.025 |

Values are given as mean±SD, no. (%), or median (interquartile range). TIA indicates transient ischemic attack; CHA2DS2-VASc, congestive heart failure, hypertension, age≥75, diabetes mellitus, prior stroke or transient ischemic attack, vascular disease, age 65–74, female; CrCl, creatine clearance; LVEF, left ventricular ejection fraction; LV, left ventricle; IV, intravenous; IA, intra-arterial; HT, hemorrhagic transformation; NIHSS, National Institutes of Health Stroke Scale.
*296 patients with transthoracic echocardiogram performed, †NIHSS < 8, ‡NIHSS 8-15, §NIHSS>15.
