# Supplementary material for: Early apixaban administration considering the size of infarction and functional outcome in acute ischemic stroke
Source: Front Neurol. 2024 Jan 26;15:1302738. doi: 10.3389/fneur.2024.1302738 (PMC10853473; doi:10.3389/fneur.2024.1302738)
Supplement: Supplementary file 2 [file Table_2.DOCX]

**Supplementary 2. Comparisons of parameters based on the size of cerebral infarction**

| Parameters | Size of the infarction | | | *P* value |
| --- | --- | --- | --- | --- |
|  | Small | Medium | Large |  |
|  | (N=118) | (N=121) | (N=60) |  |
| Female | 44 (37.3) | 51 (42.1) | 29 (48.3) | 0.361 |
| Age, years | 72.3 ± 9.6 | 75.0 ± 9.5 | 72.3 ± 11.0 | 0.072 |
| Vascular risk factor |  |  |  |  |
| Hypertension | 74 (62.7) | 85 (70.2) | 47 (78.3) | 0.095 |
| Diabetes | 27 (22.9) | 41 (33.9) | 18 (30.0) | 0.166 |
| Dyslipidemia | 38 (32.2) | 35 (28.9) | 24 (40.0) | 0.325 |
| Smoking | 44 (37.3) | 35 (28.9) | 13 (21.7) | 0.087 |
| Previous stroke/TIA | 33 (28.0) | 35 (28.9) | 16 (26.7) | 0.950 |
| Previous antithrombotics | 44 (37.3) | 52 (43.0) | 25 (41.7) | 0.655 |
| CHA2DS2-VAS_C_ Score | 5 (4–6) | 5 (4–6) | 5 (4–6) | 0.036 |
| Lab findings |  |  |  |  |
| CrCl, ml/min | 77.0 (59.0–88.0) | 71.0  (58.0–86.0) | 73.5  (57.0–91.0) | 0.574 |
| D-dimer, mcg/dl | 0.6 (0.3–1.6) | 1.0 (0.5–2.3) | 1.2 (0.6–3.1) | 0.001 |
| Echocardiographic findings^†^ |  |  |  |  |
| Left atrium size, mm | 45 (39–50) | 46 (41–50) | 43 (39–46) | 0.083 |
| LVEF, % | 61.0  (57.0–64.0) | 59.5  (54.5–64.5) | 60.0  (56.0-–63.0) | 0.407 |
| LV Wall motion abnormality | 19 (16.2) | 28 (23.4) | 11 (18.7) | 0.734 |
| Early apixaban administration | 53 (44.9) | 80 (66.1) | 37 (61.7) | 0.003 |
| HT at admission | 3 ( 2.5) | 20 (16.5) | 15 (25.5) | <0.001 |
| NIHSS severity |  |  |  | <0.001 |
| Mild^‡^ | 92 (78.0) | 59 (48.8) | 22 (36.7) |  |
| Moderate^§^ | 18 (15.3) | 41 (33.9) | 18 (30.0) |  |
| Severe^¶^ | 8 (6.8) | 21 (17.4) | 20 (33.3) |  |
| Onset to first apixaban administration, days | 4 (3–6) | 5 (3–8) | 11 (7–18) | <0.001 |
| Apixaban dose |  |  |  | <0.001 |
| 2.5 mg | 52 (44.1) | 86 (71.1) | 48 (80.0) |  |
| 5 mg | 66 (55.9) | 35 (28.9) | 12 (20.0) |  |
| Labeling dose |  |  |  | <0.001 |
| On labeling | 81 (68.6) | 65 (53.7) | 22 (36.7) |  |
| Off-labeling | 37 (31.4) | 56 (46.3) | 38 (63.3) |  |
| Favorable mRS at 3 months after stroke | 86 (72.9) | 65 (53.7) | 16 (26.7) | <0.001 |
| ERIS in 3 months | 5 (4.2) | 3 (2.5) | 5 (8.3) | 0.191 |
| Stroke progression | 1 (0.8) | 2 (1.7) | 9 (15.0) | <0.001 |
| Stroke progression or ERIS | 6 (5.1) | 5 (4.1) | 14 (23.3) | <0.001 |
| sICH in 3 months | 1 (0.8) | 1 (0.8) | 1 (1.7) | 0.847 |
| Death in 1 year | 5 (4.2) | 10 (8.3) | 2 (3.3) | 0.275 |

Values are given as mean ± SD, no. (%), or median (interquartile range). TIA indicates transient ischemic attack; CHA2DS2-VASc, congestive heart failure, hypertension, age, ≥ 75 years, diabetes mellitus, prior stroke or transient ischemic attack, vascular disease, age, 65–74 years, female. CrCl, creatine clearance; LVEF, left ventricular ejection fraction; LV, left ventricle; HT, hemorrhagic transformation; NIHSS, National Institute of Stroke Scale; mRS, modified Rankin Scale; ERIS, early recurrent ischemic stroke; sICH, symptomatic intracranial hemorrhage.
^†^296 patients underwent transthoracic echocardiogram, ^‡^NIHSS, < 8, ^§^NIHSS, 8–15, ^¶^NIHSS, > 15.
